# Atypical Cerebral Tuberculosis: Contending With the Diagnostic Challenge

**DOI:** 10.7759/cureus.110080

**Published:** 2026-06-01

**Authors:** Aishwarya Ashwinee, Sebastian Hernandez Mejia, Allen Cho, Miray Kurtca, Marutha Arulthasan

**Affiliations:** 1 Internal Medicine, Richmond University Medical Center, Staten Island, USA; 2 Internal Medicine/Cardiology, Richmond University Medical Center, Staten Island, USA

**Keywords:** cns tuberculosis, critical care, disseminated tuberculosis, infectious disease, internal medicine, medical education, medicine, public health

## Abstract

Despite effective anti-mycobacterial therapy, tuberculosis (TB) remains a major global cause of mortality, and tuberculous meningitis (TBM) is its most lethal manifestation.

We present a 61-year-old woman with no history of immunosuppression who presented with acute inattention, mild aphasia, and right-sided weakness after several days of progressive cognitive decline. She had recently traveled to Nigeria, where she was treated for malaria and typhoid fever. Initial brain imaging was unrevealing. Extensive infectious testing was inconclusive. Her condition within the first few days after hospital admission was fluctuating, with periods of transient improvement followed by clinical deterioration eventually requiring intubation on Day 5. Computed tomography (CT) scan of the brain demonstrated hydrocephalus. An empiric treatment for bacterial and viral meningitis as well as a trial of intravenous immunoglobulin (IVIG) for suspected autoimmune encephalitis yielded no improvement. On day 11, a lymph node biopsy done after a CT scan of the abdomen and pelvis revealed extensive lymphadenopathy and confirmed acid-fast bacilli (AFB), establishing disseminated tuberculosis. However, soon after this revelation, she became unresponsive, with imaging demonstrating subarachnoid hemorrhage and diffuse cerebral edema. She progressed to tonsillar herniation and eventually succumbed to the disease.

This case underscores the diagnostic challenge of TBM, often underrecognized in the modern era despite its high global prevalence and its ability to be transmitted rapidly when exposure occurs. Given the high mortality associated with delayed treatment, clinicians must maintain a high index of suspicion and consider early empiric therapy in patients with subacute meningitis and epidemiologic risk factors, even when initial testing is inconclusive.

## Introduction

Despite Robert Koch's discovery of *Mycobacterium tuberculosis* (MTB) in 1882, and the development of highly efficacious anti-mycobacterial drugs since then, tuberculosis (TB) remained the leading infectious cause of death globally before COVID-19 [1]. Tuberculous meningitis (TBM) remains the most lethal form, carrying mortality rates of 20-67% even with treatment [2].

The disease's ability to present without pulmonary involvement represents one of tuberculosis's most insidious characteristics: its capacity to hide in plain sight. In developed countries, the commonly held heuristic is that TBM mainly afflicts immunocompromised populations [2]. The incidence of TB has been steadily declining since 1992, and medical education has diminished its emphasis on tuberculosis, leaving clinicians ill-prepared to recognize atypical presentations. Additionally, non-specific clinical presentation, delayed microbiologic confirmation, and limited sensitivity across various diagnostic modalities available for tuberculosis meningitis, as seen in this case, may lead to delayed diagnosis and even irreversible disability or death.

However, recent Centers for Disease Control and Prevention (CDC) data show incremental increases in the incidence of TB in the United States(US) since 2021 to surpass pre-pandemic incidence rates in 2023 [3]. This case embodies the paradox of tuberculosis (TB) in the twenty-first century: we possess effective treatment, yet the disease thrives on diagnostic delays and the false sense of security that tuberculosis is "a disease of the past."

## Case presentation

A 61-year-old woman presents to the emergency department (ED) for an acute episode of inattention, mild aphasia, and right upper and lower extremity weakness. Reportedly, the patient had experienced a fall three days ago and episodes of inattentiveness and short-term memory loss for two to three days prior to presentation. Finally, the development of acute-onset focal weakness over several hours prompted the visit to the ED. 

The patient had no significant medical history, including no history of diabetes or alcohol use. However, travel history was significant for a trip to Nigeria three months prior, where she was reportedly hospitalized for both malaria and typhoid fever and treated with anti-malarial drugs and antibiotics. Prior to this presentation, she was alert and oriented to person, place, and time, and able to perform activities of daily living independently and worked as a home health aide. Her TB screening as part of her yearly pre-employment health requirements for the patient’s employment had been negative within the past year.

Days 1-5

During the initial encounter in the ED, her National Institutes of Health Stroke Scale (NIHSS) score was 2 (1 point for mild to moderate aphasia, 1 point for visual, tactile, auditory, spatial, or personal inattention) [4]. The patient was febrile to 102 °F (38.9 °C) with a heart rate of 114 beats per minute, meeting criteria for sepsis. Blood cultures, urine culture, sputum culture, and chest radiograph were obtained as part of sepsis workup and showed no significant findings. Empiric treatment for possible bacterial and viral meningitis was initiated with vancomycin, ceftriaxone, ampicillin, and acyclovir. Lumbar puncture (LP) was performed, which revealed moderate pleocytosis (136 white blood cells/μL), normal glucose, and markedly elevated protein (311 mg/dL), consistent with viral infection. Cerebrospinal fluid (CSF) opening pressure was 22 mm Hg. Testing for herpes simplex virus, West Nile virus, enterovirus, human herpesvirus 6, varicella-zoster virus, human immunodeficiency virus (HIV), and syphilis were all negative.

Initial computed tomography (CT) scan of the brain and CT angiography of the brain and neck showed no acute intracranial abnormalities. As the patient’s last known well time exceeded 12 hours, tissue plasminogen activator was not administered. Magnetic resonance imaging (MRI) of the brain demonstrated T2-FLAIR (T2-weighted fluid-attenuated inversion recovery) white matter changes in the left occipital, left parietal, and right cerebellar regions, suggestive of nonspecific encephalitic changes without temporal lobe involvement. Electroencephalogram (EEG) and video EEG were consistent with diffuse encephalopathy. Repeat lumbar puncture showed similar findings to prior with marginally increased opening pressure. Malaria smears (Days 2 and 4) were negative. The following table compares the findings of CSF analysis performed on Day 1 and Day 3 with reference values (Table 1).

**Table 1:** Cerebrospinal fluid (CSF) analysis of lumbar puncture (LP) on Day 1 and repeat lumbar puncture on Day 3 with reference values for comparison LP: lumbar puncture; CSF: cerebrospinal fluid; RBC: red blood cells; WBC: white blood cells; LDH: lactate dehydrogenase; PMN: polymorphonuclear cells

	Reference values	LP (Day 1)	LP (Day 3)
Color	Clear	Xanthochromic	Clear
Opening pressure	50-200 mm H2O	210 mm H2O	220 mm H2O
RBC count	Nil	9 cells/ μL	22 cells/ μL
WBC count	0-5	136 cells/ μL	182 cells /μL
WBC types	Lymphocytes	PMN, Mononuclear cells	PMN, Mononuclear cells
CSF protein	15-40 mg/dL	311.2 mg/dL	60 mg/dL
CSF LDH	<40 U/L	58 U/L	64 U/L
CSF glucose	50-80 mg/dL (two-thirds of blood glucose)	71 mg/dL	60 mg/dL
Microbial examination	No microorganisms	No microorganisms	No microorganisms

During this period, the patient’s mentation transiently improved, possibly related to two days of dexamethasone given empirically for suspected bacterial meningitis. 

Days 5-10

On Day 5, the patient developed asymmetric, involuntary upper extremity jerking movements with acute decline in mentation and Glasgow Coma Score (GCS) 6, requiring intubation for airway protection. Repeat CT brain revealed ventriculomegaly out of proportion to cortical sulci, concerning for hydrocephalus. Empiric treatment for suspected autoimmune encephalitis was initiated with IVIG. The patient completed a five-day course of IVIG but remained intubated and sedated, with persistent tachycardia (100-140 bpm) precluding weaning trials. Due to lack of neurologic improvement, on day 10, CT abdomen and pelvis (CTAP) was obtained. CT imaging revealed right upper lobe patchy opacities, extensive abdominal and pelvic lymphadenopathy (hepatoduodenal, celiac, para-aortic, paracaval, and iliac chains), and a 1 cm cystic lesion in the pancreatic tail raising suspicion for possible intraductal papillary mucinous neoplasm or lymphoma.

Days 11-13

**Figure 1:**
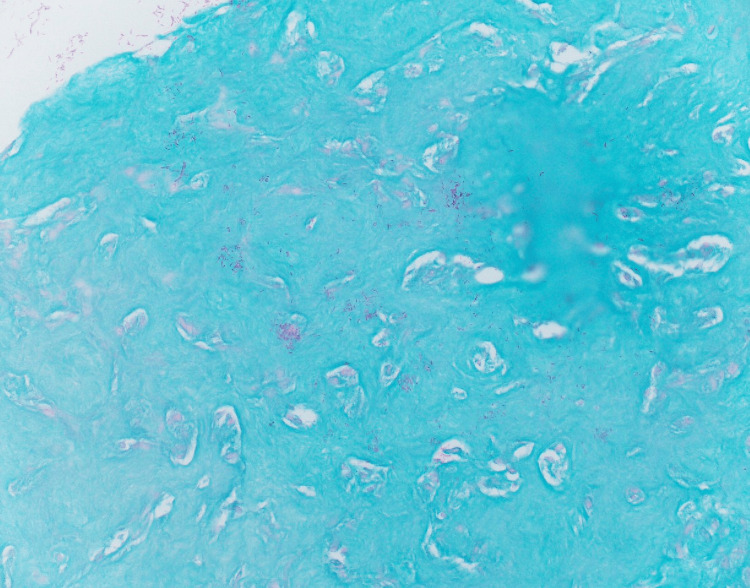
Ultrasound-guided right inguinal lymph node core biopsy Ziehl-Neelsen acid-fast stain with methylene blue counterstain, original magnification x 40, showing abundant acid-fast bacilli (AFB).

On Day 11, lymph node biopsy (Figure 1) demonstrated tuberculous lymphadenitis with abundant AFB, confirming tuberculosis. GeneXpert testing pursued on the same day also tested positive for MTB complex. Later that evening, the patient became unresponsive despite prompt initiation of anti-tuberculous treatment and escalation of the steroid regimen. Repeat CT brain showed new subarachnoid hemorrhage (SAH), diffuse cerebral edema, loss of gray-white differentiation, and a left parietal periventricular hypodensity. On Day 13, the patient progressed to tonsillar herniation.

Several days later, the patient expired despite multidisciplinary management (infectious disease, neurology, neurosurgery, critical care, and hematology-oncology). 

**Figure 2:**
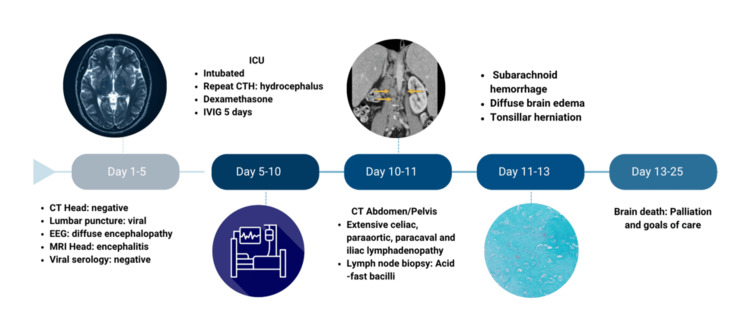
Schematic representation of the clinical progression This figure was created by the authors using Canva (Canva Pty Ltd., Sydney, Australia). CT: computed tomography; CTH: computed tomography of the head; EEG: electroencephalogram; IVIG: intravenous immunoglobulin; ICU: intensive care unit

Figure 2 summarizes the patient's hospital course with major diagnostic investigations correlated with the timeline of clinical deterioration.

## Discussion

This case illustrates the devastating consequences of delayed diagnosis in tuberculous meningitis. The patient's presentation was atypical in several respects: absence of pulmonary symptoms, negative chest radiograph, and CSF profile that initially overlapped with viral/ early TBM rather than the CSF profile classically associated with TBM. Additionally, this case challenges the heuristic that several major risk factors for immunosuppression must exist for a patient to present with fulminant tuberculous meningitis. The CSF findings of moderate pleocytosis (136 WBC/μL), normal glucose, and elevated protein (311 mg/dL) were consistent with TBM, though the normal glucose was atypical. Classical TBM typically presents with lymphocytic pleocytosis, low CSF glucose, and elevated protein [5].

The patient’s advanced age, fluctuating course with interval improvement [6], and unclear history regarding age-appropriate screening for malignancy led to the consideration of autoimmune encephalitis possibly secondary to paraneoplastic etiology, early in the hospital course. Lack of typical high-risk factors for progression to tuberculous meningitis, such as homelessness, crowded living conditions, immunocompromising conditions like diabetes, and chronic alcohol use [2], led to tuberculosis being considered lower on the differential diagnosis. However, the patient's recent travel to Nigeria and recent hospitalization in Nigeria should have raised suspicion for exposure to tuberculosis, particularly given the high TB burden in Nigeria [7]. Further, the initial clinical improvement noted with dexamethasone on Days 3-4 was consistent with the known benefit of corticosteroids in TBM [8], though this was administered empirically for bacterial meningitis rather than for TBM. The absence of pulmonary findings in this case is noteworthy, as approximately 30% of TBM patients may have normal chest imaging [9,10]; however, the development of hydrocephalus on Day 5, evidenced by dilated ventricles on CT, is a common, serious, and well-documented complication of TBM, occurring in 52-65% of adult patients [11].

Additionally, the initial presentation of focal weakness is also consistent with possible arachnoiditis frequently seen in TBM. However, due to non-specific findings on MRI and the predominant symptom being the progressive decline in mental status rather than focal neurologic symptoms, as may be expected from localizing lesions, autoimmune encephalitis and viral encephalitis were considered higher on the differential diagnoses initially. In combination with low prevalence of tuberculosis in the local population, this led to tuberculosis not being considered until other therapies had failed to show improvement. The subsequent progression to cerebral edema, subarachnoid hemorrhage, and tonsillar herniation represents the catastrophic end-stage complications of untreated TBM. The diagnosis was ultimately established by lymph node biopsy showing AFB on Day 11, but by this time, irreversible neurological damage had occurred. This case underscores that diagnostic confirmation often comes too late to prevent irreversible neurological damage and death. High rates of variability among available diagnostic modalities, demonstrated by MTB-AFB smear sensitivity rates between 10% and 50% [12], culture sensitivity of 25-70% [13], and GeneXpert sensitivity of 40-85% across pulmonary and CSF samples 4 of 6 [14,15], should discourage clinicians from waiting for confirmatory testing to guide initial treatment decisions. Early empiric treatment for TBM should be considered in such cases, as diagnostic delays are associated with mortality rates approaching 50% [16].

## Conclusions

This case demonstrates the critical importance of early recognition and treatment of TBM and highlights the importance of considering treatment empirically. Given the high mortality associated with TBM, travel to an endemic area should be considered a high-risk factor for the development of TBM. The presence of other immunocompromising conditions is not necessary for the development of TBM, as seen in this case. Subacute encephalitis and non-specific CSF findings should prompt early consideration of TBM on the differential diagnoses. Although TB is classically considered a disease of pulmonary origin due to its airborne method of transmission, due to occult systemic involvement, such as lymphadenopathy, clinical suspicion may remain low unless isolated neurologic symptoms in the absence of a clear diagnosis prompt an immediate and early association for atypical causes such as TB.

Further studies that can aggregate and analyze data from the reported cases of TBM in medical literature are necessary to establish evidence-based criteria and strong validated scoring systems - especially in developed countries, where local prevalence may be low to determine when early tuberculosis testing or therapy may be considered in cases of non-specific and atypical neurologic clinical presentation such as this one.

## References

[1] Chakaya J, Khan M, Ntoumi F (2021). Global Tuberculosis Report 2020 - reflections on the global TB burden, treatment and prevention efforts. Int J Infect Dis.

[2] Slane VH, Unakal CG (2020). Tuberculous meningitis. StatPearls [Internet].

[3] (2025). CDC. Provisional 2024 tuberculosis data, United States. Tuberculosis data. https://www.cdc.gov/tb-data/aboutprovisionaldata/2025-provisional-data.html.

[4] (2026). NIH Stroke Scale. https://www.ninds.nih.gov/health-information/stroke/assess-and-treat/nih-stroke-scale.

[5] Gupta M, Munakomi S (2024). CNS tuberculosis. StatPearls [Internet].

[6] Gole S, Anand A (2022). Autoimmune encephalitis. StatPearls [Internet].

[7] Ogunniyi TJ, Abdulganiyu MO, Issa JB, Abdulhameed I, Batisani K (2024). Ending tuberculosis in Nigeria: a priority by 2030. BMJ Glob Health.

[8] Wang W, Gao J, Liu J, Qi J, Zhang Q (2022). Clinical efficacy of dexamethasone in the treatment of patients with tuberculous meningitis: a meta-analysis. Contrast Media Mol Imaging.

[9] Thwaites GE, Van TR, Schoeman J (2013). Tuberculous meningitis: more questions, still too few answers. Lancet Neurol.

[10] Uniyal R, Garg RK, Malhotra HS (2019). Computed tomography thorax abnormalities in immunocompetent patients with tuberculous meningitis: an observational study. J Neurol Sci.

[11] Donovan J, Figaji A, Imran D, Phu NH, Rohlwink U, Thwaites GE (2019). The neurocritical care of tuberculous meningitis. Lancet Neurol.

[12] Lewinsohn DM, Leonard MK, LoBue PA (2017). Official American Thoracic Society/Infectious Diseases Society of America/Centers for Disease Control and Prevention clinical practice guidelines: diagnosis of tuberculosis in adults and children. Clin Infect Dis.

[13] Miller JM, Binnicker MJ, Campbell S (2024). Guide to utilization of the microbiology laboratory for diagnosis of infectious diseases: 2024 update by the Infectious Diseases Society of America (IDSA) and the American Society for Microbiology (ASM). Clin Infect Dis.

[14] Cresswell FV, Tugume L, Bahr NC (2020). Xpert MTB/RIF Ultra for the diagnosis of HIV-associated tuberculous meningitis: a prospective validation study. Lancet Infect Dis.

[15] Horne DJ, Zifodya JS, Shapiro AE (2025). Xpert MTB/RIF Ultra assay for pulmonary tuberculosis and rifampicin resistance in adults and adolescents. Cochrane Database Syst Rev.

[16] Donovan J, Cresswell FV, Tucker EW (2026). A clinical practice guideline for tuberculous meningitis. Lancet Infect Dis.

